# Integrated infectious disease and substance use disorder treatment for severe injection-related infections: protocol for the randomized controlled HI-SIRI trial

**DOI:** 10.1186/s13722-026-00654-6

**Published:** 2026-02-21

**Authors:** David P Serota, Daniel J. Feaster, Tyler S. Bartholomew, Shashi N. Kapadia, Lauren K. Gooden, Sean M. Murphy, Subul Malik, Teresa A. Chueng, Salma Hernandez, Allan E. Rodriguez, Elizabeth Alonso, Viviana E. Horigian, Robrina Walker, Tim Matheson, Landhing M. Moran, C Mindy Nelson, Carrigan Parish, Hansel E. Tookes, Carlos Del Rio, Lisa R. Metsch

**Affiliations:** 1 Division of Infectious Diseases, Department of Medicine, University of Miami Miller School of Medicine, 1120 NW 14th St, Suite 851, Miami FL, 33136 USA; 2 Department of Public Health Sciences, University of Miami Miller School of Medicine, Miami, FL, USA; 3 Division of Infectious Diseases, Weill Cornell Medicine, New York City, NY, USA; 4 Department of Population Health Sciences, Weill Cornell Medicine, New York City, NY USA; 5 Department of Sociomedical Sciences, Mailman School of Public Health, Columbia University, New York City NY , USA; 6 The Emmes Company, Rockville, MD, USA; 7 San Francisco Dept of Public Health, San Francisco, CA, USA; 8 Center for the Clinical Trials Network, National Institute on Drug Abuse, National Institutes of Health, Bethesda, MD, USA; 9 Division of Infectious Diseases , Department of Medicine, Emory University , Atlanta, GA USA

**Keywords:** Opioid use disorder, Stimulant use disorder, Infectious diseases, Harm reduction, Implementation science, Injection drug use

## Abstract

**Background:**

Hospitalizations for acute bacterial and fungal infections related to injection drug use are increasing in the background of the United States drug overdose crisis. These infections are a significant contributor to morbidity and mortality among people who inject drugs (PWID). Currently, limited comprehensive approaches to caring for PWID hospitalized with severe injection-related infections (SIRIs) exist. We developed a multidisciplinary model integrating infectious disease (ID), substance use disorder (SUD), harm reduction, and patient navigation treatment approaches to help improve outcomes for PWID hospitalized with SIRIs.

**Methods:**

The Holistic Intervention for Severe Injection-Related Infections (HI-SIRI) trial is a multicenter, randomized controlled trial conducted at six academic hospitals across the United States with two parallel treatment arms. Participants (*N* = 480) hospitalized with confirmed or suspected SIRI and injection drug use in the past year will be randomized to either the “SIRI team” intervention or treatment as usual (TAU) arm. Randomization will be stratified by site, primary drug (opioid vs. non-opioid), and ICU admission. The primary trial outcome is the difference in the proportion of participants alive with no hospital readmissions at 4 months post-randomization between the two arms. Secondary outcomes include SUD and ID treatment initiation, antibiotic treatment completion, all-cause and substance use related mortality, and overall healthcare utilization, assessed at 8 and 12 months post-randomization. A cost-effectiveness analysis and implementation evaluation will also be performed.

**Discussion:**

The HI-SIRI trial will be one of the first to test a comprehensive treatment approach for the syndemic of PWID hospitalized with acute bacterial and fungal infections. The trial will integrate ID and SUD treatment, built on principles of harm reduction. This innovative model, incorporating evidence-based methods, will address several multi-level barriers to care by offering low-barrier, patient-centered interventions for infection management and substance use treatment. The HI-SIRI trial seeks to transform the current standards of care for PWID hospitalized with SIRI to reduce readmissions, prevent death, and reduce healthcare costs.

**Registry:**

ClinicalTrials.gov, TRN: NCT05688423, Registration date: 17 January, 2023

**Trial Registration:**

ClinicalTrials.gov NCT05688423 (https://www.clinicaltrials.gov/study/NCT05688423). Trial registry name: CTN-0121: Integrated Care and Treatment for SevereInfectious Diseases and Substance Use Disorders (SUD) among HospitalizedPatients. Trial Sponsor: Landhing Moran, PhD, landhing.moran@nih.gov,National Institute on Drug Abuse Clinical Trials Network. Registration date:January 17, 2023. Protocol Version 4.0.

## Background

People who inject drugs (PWID) experience unacceptable, yet often preventable, harms from drug use. The most extreme harm is premature death from the medical impact of injection drug use (IDU), most commonly from overdose or infectious diseases [1]. Hospitalizations for IDU-associated infections have been increasing for the last two decades along with an ongoing drug overdose crisis [2–4]. Beyond the well-known risks of hepatitis C and HIV, severe injection-related infections (SIRIs), which include acute bacterial and fungal infections such as skin and soft tissue infections (SSTI), bloodstream infections, osteoarticular infections, and endocarditis, among others, are a significant cause of morbidity and mortality among PWID [5, 6]. Hospitalizations for these conditions typically represent the most severe end of the risk spectrum when it comes to SUD and can serve as a harbinger of mortality [7]. At the same time, SIRI hospitalizations serve as a reachable moment for PWID to engage with the healthcare system and initiate evidence-based treatments for SUD [8]. Harnessing SIRI hospitalizations by implementing the tools of harm reduction, SUD treatment, and infectious diseases care has the potential to mitigate the infectious disease/SUD syndemic.

Hospitalizations for SIRIs are characterized by prolonged lengths of hospital stay, high rates of patient-directed discharge—also known as “against medical advice,” high readmission rates, and low utilization of evidence-based SUD treatment [9–14]. Additionally, hospitalized PWID report experiencing pervasive stigma and discrimination within the healthcare system leading to avoidance of medical care, and reliance on informal self-treatment, with dangerous delays in presenting for care [15–18]. Although no comprehensive approach to the care of PWID hospitalized with SIRIs has been rigorously studied, certain interventions are critical to the foundation of a new system of care for this population [19]. First, use of medications for opioid use disorder (MOUD)—particularly buprenorphine and methadone—has been associated with reduced patient directed-discharge, increased antibiotic completion rate, and reduced mortality [20–24]. Second, a variety of antimicrobial approaches, including oral antibiotics, outpatient parenteral antimicrobial therapy, and long half-life intravenous antimicrobials can be effective for treating SIRIs [25–29]. Thus, dogmatic adherence to guidelines, which often suggest long courses of intravenous antibiotics [30, 31], is unlikely to yield favorable results for PWID; instead, a patient-centered adaptive approach is indicated. Third, the immediate post-hospital period is high risk for overdose mortality and warrants intensive implementation of naloxone distribution and low-barrier access to ongoing medical and mental health/SUD health services [32]. Finally, harm reduction interventions, such as use of sterile syringes, naloxone, and HIV prevention/treatment services are efficacious and should be implemented [33]. Abstinence from drug use is not necessarily requisite for successful infectious disease outcomes [34].

To address the often-inadequate care for PWID experiencing SIRIs, a model of integrated infectious disease, SUD, and harm reduction care was developed by the lead author at a public hospital in 2020 in Miami, Florida [35]. The Miami Severe Injection-Related Infection team (SIRI team) pilot program aimed to meet the needs of PWID hospitalized with acute bacterial or fungal infections by focusing on pragmatic antimicrobial plans, improving comfort, and helping treat SUD for those who are receptive. The team employed a harm reduction approach, provided intensive care coordination, focused on low-barrier access to MOUD, and utilized individualized ID treatment approaches, such as oral and long-acting lipoglycopeptide antibiotics. By harnessing connections with the local syringe services program (SSP), residential addiction treatment programs, and housing services, in addition to clinic and telemedicine capabilities, the team provided ID and SUD care tailored to each individual’s needs. Over the initial 24 months of the pilot, the SIRI team saw 59 patients who were followed for 90 days after discharge [9]. Among SIRI team patients, 98% of patients were discharged with MOUD (of those with OUD), 90% completed their antibiotics course, and the 90-day readmission rate was 17%, compared to 42% in historical controls [9]. Seventy-one percent were experiencing homelessness at the time of admission, and the team was able to facilitate discharge planning such that only 26% were discharged back to the street. Patients seen by the SIRI team left under patient-directed discharge in 17% of intervention patients, compared to 37% of control group patients. Qualitative work on the pilot demonstrated high satisfaction among patients and hospital providers who co-managed patients with the SIRI team [36]. Patients reported experiencing a sense of advocacy and support that they had not experienced previously within the healthcare system. Interpretation of the outcomes of the SIRI team pilot’s model is limited by the single-center observational nature of the program and its evaluation. To evaluate the efficacy and generalizability of the SIRI team model of integrated ID/SUD/harm reduction care, we developed the *Holistic Intervention for Severe Injection-Related Infections* (HI-SIRI) study.

## Methods/Design

### Study design and objectives

The HI-SIRI study is a multicenter non-blinded randomized controlled trial funded by NIDA and conducted in the National Drug Abuse Treatment Clinical Trials Network at 6 hospitals across the U.S. with two parallel treatment arms. The study aims to test the efficacy of an integrated infectious diseases (ID)/substance use disorder (SUD) clinical team intervention on improving health outcomes for PWID hospitalized with SIRIs. The SIRI team provides joint infectious diseases and addiction medicine expertise in a single care team. Participants are identified from the pool of inpatient admissions at participating hospitals. If medically stable, they are invited to provide written informed consent for a brief screening interview. If they screen eligible, provide written informed consent for full study participation, and provide HIPAA Authorization for medical record review, participants complete a baseline assessment (interview, urine drug screening, and medical record abstraction) and are randomized before hospital discharge. Randomization is stratified by 1) hospital site, 2) primary drug (opioid versus non-opioid), and 3) admission to ICU as part of the index hospitalization (ICU versus non-ICU). Participants assigned to the SIRI team meet with SIRI team members approximately daily while hospitalized and approximately weekly post-discharge over the 4-month-long intervention period. The intervention team provides care for infectious diseases and SUD, harm reduction services, patient navigation focused on SUD and social determinants of health, and general medical services throughout the intervention period. Participants assigned to treatment as usual (TAU) receive care and referrals as typically offered in the hospital setting at each site, which might include infectious disease, psychiatry, or addiction medicine consults during hospitalization. Study visits are conducted at baseline, 4 months, 8 months, and 12 months following randomization. The study protocol follows the Standard Protocol Items: Recommendations for Interventional Trials (SPIRIT) Statement (Appendix A). The study was reviewed and approved by the University of Miami Institutional Review Board (UM; IRB#20221134) which serves as the single IRB for the trial and provides regulatory oversight over all the participating sites. The study protocol is available in Appendix B.

The primary objective of the trial is to determine whether the integrated ID/SUD care team intervention (SIRI team) will be associated with lower mortality and fewer hospital readmissions compared to TAU. The primary hypothesis is that the proportion of participants alive with no hospital readmissions will be higher in the SIRI team intervention group vs. TAU group at 4 months post-randomization. Secondary objectives are to examine differences between the two study groups in important milestones that may contribute to post-discharge recovery, harm reduction, and sustained health: proportion of participants alive with no hospital readmissions at all time points (4, 8, and 12 months post-randomization) to ascertain whether differences maintain during follow-up, completion of antibiotic course, initiation of treatment of SUD by hospital discharge, receipt of outpatient care post-discharge, retention in or completion of outpatient care, and change in substance use severity and frequency. Because the primary outcome is broadly defined as no hospital readmission and being alive (compared to any hospital readmission or all-cause mortality), we will examine more detailed sources of differences between the two groups as secondary outcomes: all-cause mortality, drug use-related mortality (infections or overdose), recurrent SIRI, and hospital readmission and emergency department visit number, type, and length. Additionally, the study’s economic evaluation will estimate the cost required to implement and sustain the SIRI team intervention and determine the relative value of the intervention from the healthcare sector and societal perspectives. The study’s simulation modeling will use healthcare utilization and health outcome data to estimate the long-term clinical- and cost-effectiveness of the intervention at a population-level. The objective of the implementation evaluation component is to understand preliminary determinants and strategies that lead to successful reach, adoption, implementation, and sustainability of the SIRI team intervention.

### Setting and participants

The study site selection criteria included 1) location in a geographic area with a high prevalence of injection drug use and/or overdose, 2) census of at least 100 inpatients per year hospitalized with injection drug use-associated acute bacterial or fungal infections, and 3) prior experience participating in research/clinical studies. To maximize external validity, study sites were sought for diversity in geography, drug use patterns, and health system resources. Sites that did not have well established and robust access to SUD treatment were prioritized. The site selection committee consisted of members from the larger protocol development team. All NIDA Clinical Trial Network nodes were invited to nominate sites appropriate for the study. The selection process included three phases, which consisted of an initial survey followed by two stages of interviews of candidate sites. The included study sites are located in Miami, Florida; Tampa, Florida; Birmingham, Alabama; Salt Lake City, Utah; Albuquerque, New Mexico; and Philadelphia, Pennsylvania. The study population consists of hospitalized patients with IDU and SIRI. Individuals must meet all of the inclusion criteria and none of the exclusion criteria to be eligible to participate in the study (Box 1).

**Table:** 

Box 1. Inclusion and Exclusion criteria
In order to be eligible participating individuals must:
• be admitted to a participating hospital at the time of randomization
• be 18 years of age or older
• be experiencing a severe injection-related infection (SIRI) or suspected SIRI
• have an indication of injecting drugs in the prior year
• provide informed consent
• be able to communicate in English
• provide sufficient locator information
• sign a HIPAA form and/or EHR release to facilitate medical record abstraction
• report being willing to return for follow-up visits
Individuals will be excluded from participation if they:
• have significant cognitive or developmental impairment to the extent that they are unable to provide informed consent (or their legal guardian/representative) are unable or unwilling to give written informed consent
• are currently in jail, prison or other overnight facility as required by court of law or have pending legal action that could prevent participation in study activities
• are terminated via site principal investigator decision with agreement from one of the study lead investigators.
Note: Pregnancy is not an exclusion criterion for this study; therefore, sites may enroll pregnant women and/or follow-up with already enrolled women who become pregnant after enrollment in the study

Qualifying infections include acute bacterial and fungal infections occurring in PWID (Box 2). It can often be difficult to ascertain if an infection is directly related to the act of injecting drugs, a coincidental non-injection related infection, or infection due to exposures related to other aspects of SUD (e.g., experiencing homelessness or immunosuppression from HIV infection). For example, an individual could experience lower extremity cellulitis from injecting into veins of the leg but could also develop lower extremity cellulitis from stimulant-induced picking behaviors. We assumed that regardless of the mechanism of infection, PWID with infections experience a similar set of barriers to successful health outcomes; therefore, inclusion criteria broadly include those with one of the allowable infections among PWID. The injection drug use criterion includes self-report of injection drug use in the prior 12 months. Because injection drug use can be a stigmatized behavior, inclusion allows for other “indications of injection drug use” even if the screened individual reports no injection drug use. Acceptable indications include documented physical exam findings of injection use, classic injection-related infections (e.g. soft tissue infection in the antecubital fossa), electronic health record (EHR) chart history indicating evidence of IDU in the prior 12 months, and recent hepatitis C infection of suspected IDU origin, among other clinical indicators.

**Table:** 

Box 2. Qualifying infections for study entry
• Bloodstream infection
• Native valve endocarditis
• Prosthetic valve endocarditis
• Cardiac implantable electronic device infection
• Non-vertebral osteomyelitis
• Orthopedic hardware infection
• Vertebral osteomyelitis
• Epidural abscess
• Native joint septic arthritis
• Prosthetic joint septic arthritis
• Skin and soft tissue infection (cellulitis, skin abscess, necrotizing fasciitis)
• Septic thrombophlebitis
• Pneumonia/lung abscess/empyema
• Pulmonary septic emboli
• Biliary tract infection
• Muscle abscess/myositis
• Central nervous system infection
• Ophthalmologic infection
• Sinus infection
• Non-sexually transmitted genitourinary infection
• Other abscess
• Other acute bacterial or fungal infection deemed appropriate by site study team
• Note: all infections must be of proven or suspected acute bacterial or fungal etiology.

### Procedures and follow-up

#### Pre-screening and recruitment

Sites are recommended to implement an EHR-based pre-screening protocol to streamline screening and maximize the reach of screening procedures. This involves reviewing the daily census for indicators that a patient may be a PWID. Sites implementing EHR pre-screening are encouraged to seek a HIPAA Authorization waiver or alteration to support this activity. Once pre-screening or a direct clinician referral is complete, recruitment begins. Hospital medical staff first assess the patient’s medical stability. If the patient is stable and expresses interest, a study staff member meets them at bedside to discuss participation. Recruitment in the hospital environment presents challenges—patient acuity, rapid care pace, off-unit testing, and frequent interruptions. To address this, interviewers are trained extensively on hospital workflows, engage with staff, and learn strategies to negotiate interview time and space. Where permitted, IRB-approved recruitment materials (e.g., flyers, brochures) are left in sealed envelopes in a patient’s room if they are unavailable. Trained research staff will complete screening, obtain informed consent (Appendix C), and conduct all study assessments.

#### Measures and timing of follow-up assessments

Data collection occurs at screening, baseline and at 4-, 8- and 12-month follow-up visits (Table 1). All participants complete quantitative assessments via computer assisted personal interviews (CAPI) through a secure Web-based platform, Advantage eClinical©. The CAPI takes approximately 2 hours to complete and includes measures of socio-demographics (e.g., basic demographics, housing instability, food insecurity, neighborhood characteristics); comorbid conditions; health services utilization; mental health and social support; physical abuse and interpersonal violence; self-efficacy, resilience and self-esteem; perceived stigma, medical mistrust and discrimination in medical settings; care team/participant relationships; health literacy; access to care and barriers to treatment; criminal and legal activities; and PhenX Tier 1 measures adopted across NIDA-funded research studies (e.g., educational attainment, employment status, marital status, body mass index, quality of life, substance use history and practices). Substance use frequency (ASI-lite assessment, including route of use), problematic alcohol use (*Alcohol Use Disorders Identification Test;* AUDIT), and drug use patterns and severity of problems related to drug use (*Drug Abuse Screening Test;* DAST-10) will be collected at all visits.

**Table 1:** Table of assessments

Assessment	Visit				
	Screening	Baseline	4 Month Follow Up	8 Month Follow Up	12 Month Follow Up
**Interventions**					
SIRI team					
Treatment as usual					
**Screening**					
Demographics	X				
Housing Instability	X		X	X	X
**Baseline and Follow-up**					
Health Literacy		X			X
Access to Care		X	X	X	X
Non-study Medical and Other Services		X	X	X	X
Perceived Barriers to Treatment		X			
Care Team-Patient Relationship		X	X	X	X
PROMIS PROPr 29 + 2		X	X	X	X
Discrimination in Medical Settings		X	X	X	X
Group-Based Medical Mistrust		X	X	X	X
Alcohol and Substance Use History		X	X	X	X
CTN-ASI: Drug/Alcohol Use Modified		X	X	X	X
Drug Abuse Screening Test		X	X	X	X
The Alcohol Use Disorders Identification Test		X	X	X	X
Alcohol and Drug Use Practices		X	X	X	X
Overdose Information		X	X	X	X
Subjective Opiate Withdrawal Scale		X			
Drug Craving Scale		X	X	X	X
Risk Assessment Battery-Part I: Needle Use		X	X	X	X
Readiness for Drug Treatment and Negative Attitudes		X	X	X	X
Perceived Stigma of Addiction Scale		X	X	X	X
Self-Report of Hepatitis/HIV Testing		X	X	X	X
Generalized Self-Efficacy		X	X	X	X
Connor-Davidson Resilience Scale		X			X
Quality of Life Enjoyment and Satisfaction		X	X	X	X
Short Social Support Scale		X	X	X	X
Rosenberg Self-Esteem Scale		X	X	X	X
Household Food Insecurity Access Scale		X			X
Criminal and Legal Activities Form		X	X	X	X
Physical Abuse and Interpersonal Violence		X			X
Concise Health Risk Tracking – Self Report Suicidal Behavior Evaluation		X	X	X	X
SIRI team Satisfaction Survey			X		
**Labs**					
Urine Drug Screen		X	X	X	X
**Targeted Safety Events**					
ED Visit and Hospitalization Form	To be completed for every ED Visit and Hospitalization that occurs after randomization and discharge from the index hospitalization; entry into the data system required within 48 hours of site awareness				

ED, emergency department; HIV, human immunodeficiency virus; SIRI, severe injection-related infection

Additionally, the baseline CAPI encompassed completion of a detailed locator form that helps study personnel to remain in contact with participants and locate them to remind them of follow-up visits; the form will be reviewed and updated (as applicable) at each follow-up visit.

All participants complete HIPAA Authorization forms for the index/participating hospital system and (as needed) medical records release forms for other health systems/facilities accessed during study participation. Medical records abstraction occurs throughout the study as needed and up to approximately 14 months post-randomization to ascertain study outcomes (e.g., hospital re-admission, diagnoses) and corroborate self-report of health services utilization.

#### Compensation

Participants are compensated for participating in study visits/activities, not for interacting with the SIRI team. Compensation is in accordance with the IRB of record’s policies and procedures, subject to IRB approval and consistent with local standards. Suggested compensation is $5 for the screening interview, $50 for each of the baseline and follow-up visits and $5 for updating locator information before each of the follow-up visit.

#### Retention

Completion of a locator form (which solicits detailed contact information for participants, their physical description, and detailed contact information for at least one contact person, e.g., relative, friend, case worker) along with providing minimal compensation for updating the locator form between study visits is critical to retaining participants. Participating sites hired an outreach worker who is responsible for relaying study visit reminders via appointment reminder cards, phone calls, text messages and/or other mechanisms as detailed in the locator form and who will track participants’ whereabouts on an ongoing basis. This includes performing street outreach visits to participants’ residences and hangouts as needed. When a participant is unreachable, the outreach worker checks the local jail log(s), prison log(s), medical records, and local coroner’s office to determine the participant’s disposition. Additionally, the outreach worker facilitates transportation to study visits, as needed, by driving participants and/or organizing and facilitating public transportation. To aid the outreach worker with identifying participants in the field, sites may obtain participant authorization to take a reference photograph of the participant. All photographs will be destroyed when the study is completed. To further facilitate retention, study participants may be issued a mobile phone (including approximately 12 months of service) at or after randomization.

#### Allocation concealment and blinding

Study staff enrolling participants at bedside are responsible for completing the baseline assessment, confirming eligibility with a site principal investigator, and informing participants and the intervention team to which arm they have been randomized. Allocation concealment will be achieved by the use of a computer-based randomization mechanism. Stratification factors across 6 different sites will further obscure any ability to predict allocation. Because the intervention is a clinical team intervention, participants cannot be blinded to receipt of the intervention. Study staff conducting follow-up assessments will not be blinded to treatment assignment.

### Intervention: SIRI team arm

Participants randomized to the intervention receive integrated infectious disease and SUD care (SIRI team) both during the hospitalization and after hospital discharge for 4 months post-randomization. The intervention is based upon six general principles for treating PWID with infectious complications and is informed by harm reduction.

1. *Medications for SUD as integral to management of infectious complications* [20–22, 25, 37–39]– The intervention team will offer to initiate and continue/maintain medications for OUD and other SUDs in a low-barrier fashion, as indicated in standard clinical care, with a focus on harm reduction. If the participant accepts the medication, the team will facilitate inpatient initiation of medications as well as manage these treatments in the post-hospital intervention period.
2. *Integration of ID and SUD care* [9, 40–42]– A single team provides consultation for both ID and SUD care for hospitalized participants. During the hospital stay, this promotes holistic and realistic decision-making regarding infectious disease treatment plans. After discharge, the team serves as a one-stop-shop for addressing participant medical and social needs, with a focus on ensuring the infection is being treated and the harms of substance use are mitigated.
3. *Longitudinal care with familiar providers* [36, 43, 44]– Medical mistrust is common among PWID due to near-universal experience of stigma and discrimination in the healthcare system. The same specialized treatment team, experts in providing sensitive and culturally competent care to PWID, follow participants from the hospital setting and beyond into the community. The team serves as the ID and SUD treatment service both during and after hospitalization.
4. *Multidisciplinary care and care coordination* [45–48]– Hospital medical, nursing, and case management teams are often inexperienced in caring for PWID and helping to meet their unique needs. The intervention team serves an important role in coordinating care, ensuring appropriate treatment of participants, and helping facilitate therapeutic discharge plans.
5. *Tailored antibiotic options and care settings* [25, 28, 29, 49, 50]– The team chooses from antibiotics that are considered standard clinical care but may decrease the need for extended hospitalization or utilization of peripherally inserted central catheters. The treatment team ensures the best antibiotic plan for each individual in the context of the available ID literature, while taking into account the individual’s substance use and social circumstances.
6. *Harm reduction* [33, 34, 51–56]- Many patients hospitalized with SIRIs do not want to cut down or stop using drugs, and for those that do, complete abstinence can be difficult to achieve. Applying harm reduction to the care of patients with SIRI is critical to facilitating complete infection treatment and avoiding preventable drug-associated morbidity and mortality. Harm reduction informs how the intervention is delivered as well as the outcomes measured.

#### SIRI team intervention team members

The SIRI team is comprised of physicians, advanced practice providers, and patient navigators. The study physician role—which may be fulfilled by multiple physicians (infectious disease, internal medicine, addiction medicine)—manages the intervention team and leads clinical care for participants. The physicians are experienced in managing infectious diseases as well as SUDs. The physicians are heavily involved in initial consultations, key treatment decisions, and follow-up care, as needed, and supervising the advanced practice providers (i.e., nurse practitioner/physician assistant role. The advanced practice provider team member manages medical aspects of inpatient and outpatient care. This includes coordinating care in the hospital between primary teams, case managers, nurses, and other specialists. The advanced practice provider manages post-hospital care with oversight from the physician and assistance from the patient navigator. Patient navigators are individuals with behavioral health, social work, case management, or other counseling experience. Patient navigators help assist intervention participants with any services they may need to optimize their infection and SUD outcomes. This includes helping to secure IDs, complete paperwork for medical insurance, housing, and SUD treatment, provision of referrals, interaction with family members, appointment reminders, reinforcement of the medical team’s recommendations, assistance with accessing medications, and general support. Patient navigators may have lived experience with SUD to enhance their ability to provide culturally competent care for participants.

#### SIRI team inpatient care

After randomization to the intervention group, participants receive the clinical services of the SIRI team for up to 4 months. The SIRI team evaluates the participant and provides consultatory care for participants in concert with their primary medical or surgical team. Infectious disease care includes assistance in diagnosis and management of the qualifying acute bacterial or fungal infection. In contrast to a typical ID consult service, the SIRI team will assist in management of SUD. This integration includes performing a substance use history, evaluation, diagnosis of SUDs, and recommendations for management of SUD, based on the participant’s willingness (or readiness) to manage their substance use. The SIRI team will also be involved with managing other symptoms associated with SUD and acute infections including pain and anxiety. Due to individuals’ acute medical needs, participants may have already received consultative services from infectious disease or addiction medicine physicians before they enroll in the study. In these cases, the intervention team will discuss with both services to facilitate a warm handoff of care to the SIRI team.

The SIRI team employs an inpatient harm reduction protocol to screen, treat, and prevent other infectious diseases common among PWID. The team also aims to reduce overdose by ensuring access to naloxone. Participants living with HIV are offered inpatient initiation of anti-retroviral therapy (ART). For participants with chronic hepatitis C, all baseline labs and studies are obtained, as part of usual clinical care, to facilitate treatment in the outpatient setting. Participants with indications for hepatitis A or B vaccination are offered these vaccines during the hospitalization. Participants without HIV infection will be educated on pre-exposure prophylaxis (PrEP). For those interested, PrEP is prescribed upon hospital discharge or they may be navigated to another local PrEP provider.

Other inpatient services provided by the SIRI Team that fall outside the standard of care for most typical hospital-based ID consult services include the following: 1) Close coordination and frequent communication with hospital case manager/social worker to help guide discharge planning; 2) working to identify funding sources for post-hospital medications and follow-up services; 3) facilitating access to a box of intranasal naloxone immediately before or after discharge; 4) prescribing necessary post-discharge medications, pick-up from pharmacy, and delivery to participants.

If a participant is readmitted to the same hospital during the 4-month intervention period, the intervention team will re-engage with them in the hospital and again provide SIRI team care. If a participant is admitted to a different hospital during the intervention period, the SIRI team will attempt to coordinate with physicians in the non-index hospital to help coordinate post-hospital care. Similarly, while SIRI team provider will not be able to directly provide care to participants while incarcerated, they may attempt to advocate for individuals and communicate with providers in the department of corrections.

#### SIRI team post-discharge care

After hospital discharge, the SIRI team continues to care for participants until 4 months after randomization. Based on the median length of stay, the expected post-hospital period will be at least 3 months. In addition to their role as a care navigator and counselor, the patient navigator serves to facilitate low barrier communication with the medical providers. Meetings may occur in person as formal clinic visits, drop-in clinic hours, videoconference, telephone/text messaging, or “home” visits by the patient navigator.

Key evidence-based harm reduction interventions include overdose education and naloxone distribution, referral to SSPs (if available), discussion of safer injection practices, navigation to PrEP services, and low barrier access to MOUD [33, 51]. For participants with OUD, the team manages MOUD directly and helps ensure linkage to methadone programs for those utilizing methadone. The SIRI team works to identify local substance use treatment programs, including those offering contingency management for stimulant use disorder. If local or app-based contingency management programs are available to participants, the SIRI team will help navigate participants to these programs as part of standard clinical care.

The SIRI team will provide care in whichever modality is feasible throughout the intervention period. This may include traditional clinic visits, street medicine, home visits, visits to residential addiction treatment, and telehealth encounters, among others.

#### Patient navigator component

The patient navigator approach includes five functions: 1) establishing an effective working relationship with the participant; 2) encouraging identification and use of strengths, abilities, and assets; 3) supporting participant control over goal setting and the search for needed resources; 4) viewing the community as a resource and identifying informal sources of support; and 5) conducting case management as an active community-based activity.

The patient navigator goals are to assess and assist participants in four primary domains: 1) Medical: Patient navigators facilitate participants completing antibiotic therapy for their acute infection and help participants meet other medical needs, such as getting to medical appointments, managing medications, and encouraging adherence to treatments. 2)

Substance use: Patient navigators use motivational interviewing techniques to ascertain barriers and facilitators to reducing substance use. They ensure medical treatment for SUD by the medical providers, according to participant desires. They assist with linking to other community substance use and/or harm reduction programs (residential treatment, intensive outpatient, SSPs, etc.). Patient navigators will also conduct outreach to locate missing participants—including homeless encampments and shelters—to continue to provide services. For participants who continue drug use, the patient navigator promotes harm reduction and helps facilitate safer drug use. 3) Housing: For participants experiencing homelessness, patient navigators actively connect participants with local shelters and housing programs and food resources. 4) Access to medical care: patient navigators help participants obtain access to health insurance and other funding sources for ongoing care, especially in the post-intervention period.

#### Social determinants of health

The SIRI team approach to care inherently considers the social determinants of health to mitigate these barriers to meet participants’ needs. Each site’s participants experience different barriers due to unique social, demographic, political, and cultural environments.

#### Post-intervention infectious disease and SUD treatment linkage to care

The SIRI team works with participants to identify and link to post-intervention care. The medical providers determine if participants require follow up infectious disease specialty care for their acute infection. Other common conditions requiring long-term care include SUD, HIV, and hepatitis C, in addition to general primary care needs.

### Control: treatment-as-usual arm

Participants assigned to the TAU arm receive the standard treatment for their SIRI and SUD at each hospital. While TAU may differ between sites, it is typically comprised of a participant cared for primarily by a hospital medicine physician with consultation by infectious diseases and either psychiatry or an addiction medicine physician. If the ID or addiction teams believe post-hospitalization follow up is indicated, each service will follow local protocols for arranging post-discharge continuation of care. This standard of care typically consists of providing participants with an appointment for follow up in an ambulatory care clinic or a list of options for follow-up treatment. Post-discharge care is sometimes provided by the same clinician seen during the hospitalization, but other times it is a colleague or different care team in the community. ID and SUD care are usually provided separately, though some sites may have more novel approaches to co-localized treatment after hospitalization. Some primary care physicians are capable of providing follow-up ID and SUD treatment. Study visits for participants assigned to TAU will include documentation of how care was delivered during the hospitalization and in the post-discharge period.

### Intervention discontinuation and cross-over

The intervention continues until 122 days post-randomization. Because the intervention includes active monitoring of participant medical records and ongoing outreach, the intervention will be terminated early only if a participant withdraws consent from the study or dies prior to day 122. Control group participants are not treated by the SIRI team intervention, as outlined in the intervention manual; however, they are permitted to be treated by clinician members of the SIRI team in non-SIRI team contexts. For example, a SIRI team physician is permitted to be the outpatient HIV provider for a control group participant, as long as the care provided falls within the usual treatments provided by that physician and clinic, outside of the context.

### Outcomes

The primary outcome is a binary measure: a participant is alive with no hospital readmissions 4 months (122 days) post-randomization OR a participant has died or been readmitted to the hospital within 4 months post-randomization. The primary outcome measure is the proportion of those who are alive with no hospital readmission at 4 months post-randomization in the SIRI team intervention compared with TAU. Hospital readmission is defined as either observation or inpatient status in an acute care hospital after discharge from the index hospitalization (i.e., the hospitalization during which randomization occurred). Elective hospital admissions, for example, for planned chemotherapy or elective surgeries, are excluded. Hospital admissions for delivery of a child in pregnant participants will be included as elective admissions. Admission to behavioral health hospitals, medically supervised withdrawal units, or psychiatric-only admissions are also excluded. The primary outcome was chosen to be relevant to patients and healthcare systems. Healthcare utilization outcomes, such as readmission and emergency department visits, will be measured using both EHR data and self-report, confirmed by EHR. At baseline assessment and each follow-up study visit, participants will be asked about healthcare service utilization and if they report receipt of services, a release of information for each relevant institution will be obtained and records requested to confirm self-report. All participants will have the EHR of the study site (*index hospital*) healthcare system queried for health services use. Additionally, any integrated health information exchange services, such as Care Everywhere for Epic, will be queried. Deaths will be determined from EHR data, local medical examiner databases, and information from emergency/secondary contact persons for participants who cannot be located. The secondary outcome related to the primary outcome of the study includes being alive and free from readmission across all study time points (4, 8 and 12 months). Other secondary outcomes are listed in Table 2.

**Table 2:** Key secondary outcomes

Outcome	Measure	Time point
Completion of planned antibiotic course	Combination of medical record abstraction and self-report	Assessed at 4-month visit
New or recurrent SIRIs	Combination of medical record abstraction and self-report	4, 8, and 12 months
Non-fatal overdose	Combination of medical record abstraction and self-report	4, 8, and 12 months
Mortality All cause •All cause •Substance use-related (SIRI, overdose)	Combination of medical record abstraction and other sources (e.g., coroner’s report)	Continuous
Substance use severity and frequency	Drug Abuse Screening Test, Alcohol Use Disorders Identification Test, and Addiction Severity Index	4, 8, and 12 months
Initiation of SUD treatment before hospital discharge •MOUD •MAUD	Medical record abstraction	n/a
Initiation of ID treatments before hospital discharge • ART/PrEP • HCV DAAs • Immunizations	Medical record abstraction	n/a
Receipt of post-discharge SUD treatments •MOUD •MAUD •Intensive outpatient •Mutual support •Residential treatment	Combination of medical record abstraction, self-report, and urine test for methadone and buprenorphine	4, 8, and 12 months
Receipt of post-discharge infectious disease treatments	Combination of medical record abstraction and self-report	4, 8, and 12 months
Service utilization measures •Length of stay •Patient directed discharge •Readmissions (count and binary) •ED visits (count and binary)	Combination of medical record abstraction and self-report	4, 8, and 12 months

ED, emergency department; HCV DAAs, hepatitis C virus direct acting antivirals; HIV, human immunodeficiency virus; MAUD, medication for alcohol use disorder; MOUD, medication for opioid use disorder; PrEP, HIV pre-exposure prophylaxis; SIRI, severe injection-related infection; SUD, substance use disorder

### Implementation evaluation component

In accordance with a hybrid type I implementation-effectiveness approach, we will employ a mixed-methods implementation evaluation utilizing the Practical, Robust Implementation and Sustainability Model (PRISM) [57, 58]. The primary objective of the implementation evaluation is to understand the implementation determinants (i.e. barriers and facilitators), contextual factors and corresponding implementation strategies that support successful reach, adoption, implementation, and sustainability of the SIRI team intervention.

Implementation outcomes will be evaluated by conducting qualitative participant exit interviews (post-intervention only) and key informant interviews (pre- and post-intervention implementation). Specific EHR data (e.g., socio-demographics, co-morbidities) will also be included in the implementation analyses to assess the characteristics of participating patients. Outcomes include: reach (e.g., number of those eligible who engage in the study, baseline comparisons between those who decline versus those who accept participation), effectiveness at the patient level (i.e., the primary outcome), adoption at the staff level (e.g., number and proportion of physicians who agree to refer patients to the study, barriers to staff-level adoption), implementation (e.g., fidelity to the intervention manual, implementation cost analysis) and maintenance (e.g., cost-effectiveness, budget impact/cost savings, facilitators of long-term intervention use).

Participant exit interviews will be conducted with participants from both the intervention and control groups at all 6 sites, with anticipated samples of 10–15 participants per study group (approximately 20–30 participants per site). These brief exit interviews (approximately 30 minutes) will occur at the conclusion of each participant’s intervention period in a private room by a trained study team member at each individual site. These designated site interviewers will be trained by the Implementation team Co-Investigators prior to interview initiation and will follow the interview guide. To capture the PRISM domain of “Patient Perspective (Receptiveness to SIRI Team, Satisfaction),” guide items include participant’s satisfaction with the SIRI team plus any considerations related to intervention adaptation. All participants will receive compensation upon completing the interview (suggested amount is $50, but the amount and method, e.g., gift card/cash, will be determined based on local standards).

Key informant interviews will be conducted with a sample of 36–48 key informants (6–8 per site) at two time points: 1) prior to study launch and 2) post-intervention (near the time that the intervention period has closed for the last participant at a given site). Given the objective of gathering qualitative data to examine barriers to staff-level adoption, key informants include any study personnel deemed critical to the intervention development and implementation, such as physicians, nurse practitioners, physician assistants, and case managers. Interviews will be scheduled based upon key informant schedule and availability, and interviewees will be consented to participate in both qualitative interviews prior to initiation. The interviews are brief (approximately 45–60 minutes) and open-ended. One of the Implementation team co-investigators conducts the interviews either by teleconference or videoconference (e.g., Zoom). All participants receive an Amazon gift card in the amount of $50 upon completing the interview.

### Economic evaluation

The trial includes a comprehensive economic evaluation to estimate the cost and cost-effectiveness of the SIRI team intervention relative to TAU from the healthcare sector, state policymaker, and societal perspectives. The primary outcome for the economic evaluation is the incremental cost-effectiveness ratio (ICER), which is defined as the incremental mean cost of the SIRI team intervention relative to TAU divided by the incremental mean effectiveness [59]. Program costs will be determined using a microcosting approach and identifying the resources used by sites to implement and sustain the intervention, through site visits, interviews with program staff, and review of study records [60, 61]. Participant data will be measured through electronic health record data collected on case report forms, as well as self-reported data collected via the Non-study Medical and Other Services (NMOS) instrument [62–66]. Costs will be estimated using the resource-costing method, wherein each resource unit consumed/utilized will be weighted by a nationally-representative, inflation-adjusted dollar value intended to reflect the real-world costs faced by decision-makers aligned with each perspective.

Three measures of effectiveness will be utilized in the cost-effectiveness analysis. The primary measure of effectiveness is the quality-adjusted life-year (QALY). Health-related quality of life (HRQoL) will be measured at each time point using the Patient-Reported Outcomes Measurement Information System (PROMIS)-Preference (PROPr) instrument. We will additionally evaluate opioid-free years, a measure of opioid use frequency, which has long been used in economic evaluations of OUD care [67, 68]. As the HI-SIRI trial also includes participants without OUD, we will also utilize a similar measure of time free from IDU. These measures will be constructed using a combination of urine drug testing results and participant self-report.

### Statistical analysis

#### Sample size and power analysis for efficacy outcome

Effect size was estimated for fixed power and sample size using PASS 2020. Since the true proportion of success in each treatment group is unknown, effect size was calculated for 80% power, a sample size of 480, and varying proportions of success in the TAU treatment group from 0.35 to 0.70 in steps of 0.05. Effect size was estimated using a logistic regression model for a binomial outcome with a logit link. The stratification factors used at randomization (site, type of primary drug use and admission to ICU as part of the index hospitalization) were not included in the power calculation model and therefore the power estimates are conservative. However, the stratification factors will be included in the analysis model at the analysis stage as fixed effects to improve the precision of the estimates. Table 3 shows the estimated effect size for varying proportions of success in the TAU treatment arm. The resulting risk differences and odds ratios corresponding to the proportions of success are also included for interpretation of the varying effect sizes. For a sample size of *N* = 480, there is 80% power or more for an absolute risk difference of 12.8% regardless of the level of success in TAU.

**Table 3:** Power calculations for primary outcome based on potential rates of success in treatment as usual group

Sample Size	Power	Proportion of Success in		Risk	Odds
		TAU	SIRI team	Difference	Ratio
480	0.80	0.350	0.476	0.126	1.684
		0.400	0.527	0.127	1.672
		0.450	0.578	0.128	1.669
		0.500	0.627	0.127	1.678
		0.550	0.674	0.124	1.695
		0.600	0.721	0.121	1.721
		0.650	0.766	0.116	1.764
		0.700	0.810	0.110	1.825

SIRI, severe injection-related infection; TAU, treatment as usual

#### Analyses of primary and secondary outcomes

A logistic regression model will be employed: alive and no hospital readmission (excluding elective treatment/procedures, detoxification-only or psychiatric admissions to a specialty hospital) OR any hospital readmission or death since previous visit as implemented in SAS. The test of the primary hypothesis will be achieved using the Wald test associated with the coefficient on the treatment indicator with the outcome at the 4-month follow-up. The model will include fixed effects for treatment group, site, type of primary drug use (opioid versus non-opioid), and admission to ICU as part of the index hospitalization (ICU versus non-ICU). The primary outcome hypothesis that the proportion of participants alive with no hospital readmissions will be higher in the SIRI Team intervention group vs. TAU group at four months post-randomization will be evaluated using a two-sided test with an a priori alpha (i.e., type I error) of 0.05. Since odds ratios can exaggerate interpretation of relative risk, particularly with larger incidence of the outcome, a Poisson distribution with log link and robust standard errors may also be considered to estimate the risk ratio directly.

For the primary outcome, data lost to missed visits will be supplemented via external records. Confirmation of death from any cause or of participant hospital readmission from the EHR abstraction will be considered a failure. If a participant is lost to follow-up and no records (EHR, medical examiner, emergency contacts’ report) are found to show death or readmission for the duration of the study, the outcome will be considered a success. Missing data is only anticipated for 1) participants who withdraw from the study up to and including study day 122 and who have not signed the consent addendum expressly permitting abstraction to continue, or 2) participants who withdraw from the study at any time during the follow-up period AND who have not signed the consent addendum AND EHR abstraction has not been performed up to and including study day 122 prior to the participant’s withdrawal. At baseline, participants will sign releases for all local health systems that they typically visit. These records will be supplemented by a query of EHR integration services, such as CareEverywhere in Epic Systems. For participants who miss their 4 month visit but attend the 8 or 12 month visit, they will be asked about readmissions dating back to the time of randomization.

A secondary analysis of the hospital readmissions across all time points will be conducted. For this analysis, a Generalized Estimating Equations (GEE) model will be employed with a binomial distribution and logit link as implemented in SAS for the secondary outcomes of proportion of participants alive with no hospital readmissions utilizing all time points. A GEE model is used to control for correlation associated with repeated measures within an individual since the analysis will be a longitudinal one at 4-, 8-, and 12-months. The model will include fixed effects for treatment group, time, the treatment-by-time interaction, site, type of primary drug use (opioid versus non-opioid), and admission to ICU as part of the index hospitalization (ICU versus non-ICU)). The secondary outcome hypotheses are that the proportion of participants alive with no hospital readmissions will be higher in the SIRI Team intervention group vs. TAU group at eight months (and, separately, at twelve months) post-randomization). These two hypotheses will be evaluated each using a two-sided test with an a priori alpha (i.e., type I error) of 0.05.

Additional secondary analyses will utilize the methods for the primary analysis if they are a single dichotomous outcome. If they include repeated measures, they will follow the analysis strategy of the above secondary outcome description.

#### Implementation evaluation analyses

With participants’ consent, all interviews will be audio-recorded and transcribed verbatim for thematic analysis. The audio recordings will then be sent to a transcription company for transcription for data analysis purposes. Transcript data will be analyzed using an iterative, inductive approach drawing on primarily content analysis. One member of the research team will read a subsample of interviews and will develop a provisional coding scheme to identify what the participants perceived to be concerns or facilitators related to their participation, at the participant-level or the key informant-level. A second research team member will review a different subsample to validate the adequacy of the provisional coding scheme as well as identify any additional codes that may be appropriate. Categories will be reviewed and any determined to be conceptually overlapping will be collapsed. Subsequently, using a finalized coding scheme, all interviews will be independently coded by two research team members. Kappa will be calculated to measure inter-rater agreement on the coding of the interview transcripts. Text relevant to code will be excerpted for analysis and interpretation by the research team and described according to the PRISM domains and constructs. We will use a software program for qualitative analysis of textual data (e.g., NVivo).

#### Economic evaluation analyses

Multivariable regressions within a generalized linear mixed model (GLMM) framework will be used to generate predicted mean values for each resource/cost category, as well as each effectiveness measure. We will model the person-period over the duration of the intervention and follow-up periods. The final predicted mean values will be estimated via the statistical method of recycled predictions, and standard errors will be derived from non-parametric bootstrapping techniques [61]. QALYs gained will be estimated using the predicted utility values from the PROPr and the area-under-the-curve method, while other effectiveness and cost measures will be summed and tested over the intervention phase, as well as the entire observation period. The predicted mean values for each phase will be used to generate ICERs for each of the 3 effectiveness outcomes and each of the 3 perspectives. Cost-effectiveness acceptability curves will then be created to assess the uncertainty surrounding each ICER, as they illustrate the probability that the intervention is cost-effective across a range of value thresholds that reflect a decision-maker’s willingness-to-pay for a unit of effectiveness. One-way sensitivity analyses will be conducted to assess uncertainties related to assumptions, parameter estimates, and model specifications.

### Oversight and management

#### Composition of the coordinating center and trial steering committee

The trial Clinical Coordinating Center (CCC) and Data and Statistics Center (DSC) will be The Emmes Company. The trial steering committee will include lead investigators, the national project director, the CCTN scientific officer, members of the CCC and DSC, and other members of the data, monitoring, and compliance teams. The steering committee will meet weekly to discuss trial progress, protocol deviations, and other matters

#### Composition of the data monitoring committee, its role and reporting structure

The independent CTN Data and Safety Monitoring Board (DSMB) will regularly review accumulating safety and efficacy data to ensure participant protection while meeting the study’s scientific objectives. The DSMB is responsible for conducting periodic evaluations to determine whether the trial should continue as planned, be modified, or be halted. Decisions may be based on concerns related to participant safety, evidence regarding the efficacy of the intervention, or issues with trial performance, such as inadequate recruitment. Reports summarizing safety and efficacy findings will be prepared for DSMB meetings at a frequency of no less than once per year.

#### Adverse event reporting and harms

This low-risk study will not involve investigational drugs or devices, so no causally related adverse events (AEs) or serious adverse events (SAEs) are expected or will be monitored. Safety reporting will focus only on targeted safety events (TSEs): emergency department visits, hospitalizations, overdoses, suicidal ideation, and deaths. These will be documented on designated forms and entered into the Advantage eClinical system. The Clinical Coordinating Center will report TSEs to the NIDA and the DSMB. All study sites have established procedures for managing medical and psychiatric emergencies, which staff will continue to follow. Site treatment providers will monitor participants for clinical deterioration or other concerns and take appropriate action when necessary.

#### Plans for communicating important protocol amendments to relevant parties

Any amendments to the protocol or consent will be approved by the IRB prior to implementation. Participants will be reconsented if the consent is amended to include new risk information (e.g., changes in the anticipated risks/benefits of participation) or other information that involves significant changes to the procedures of the study. Protocol updates will be posted to clinicaltrials.gov.

#### Data collection, quality, and management

A centralized DSC oversees data management, including developing and validating eCRFs, the study database, and training site staff. Advantage eClinical is used for data entry, and QualtricsXM for SIRI team member assessments. Sites enter accurate, complete data from source documents into eCRFs by authorized staff, following DSC guidelines. The DSC provides data dictionaries, user guides, and instructions, and conducts ongoing validation, discrepancy checks, and audits comparing source data to eCRFs. Queries are issued for missing or inconsistent data, and progress/quality reports posted daily to a secure site. Participant data uses unique study IDs and systems are password-protected. Upon completion, the DSC will clean, lock, and archive datasets, providing both raw and de-identified versions to NIDA. Training covers assessments, eCRF use, and quality standards, with data monitored to meet predefined quality thresholds.

#### Data sharing and dissemination

This study will comply with the NIH Data Sharing Policy and Implementation Guidance. Investigators will register and report results of the trial in ClinicalTrials.gov, consistent with the requirements of the Policy on the Dissemination of NIH-Funded Clinical Trial Information and the Clinical Trials Registration. Primary data for this study will be available to the public in the NIDA data repository, per NIDA CTN policy. The primary outcome publication will be included along with study underlying primary data in the data share repository, and it will also be deposited in PubMed Central, per NIH Policy. The planning, preparation, and submission of publications will follow the policies of the Publications Committee of the CTN.

#### Confidentiality

Participants will provide written informed consent and complete HIPAA Authorization and/or EHR release forms. Only authorized personnel will have access to identifiable data, which is encrypted and stored securely. IRBs, regulatory agencies, and sponsors may inspect records for compliance. Participants will be assigned unique study IDs. Records will be retained for at least 3 years post-study, or longer per IRB/state/federal requirements.

## Discussion

The HI-SIRI study will serve as a major advancement in addressing the complex health needs of PWID suffering from infectious diseases. If the intervention is proven effective in reducing the rate of readmissions and mortality, the HI-SIRI study could transform the treatment of PWID experiencing SIRIs.

The pilot program conducted by the Miami SIRI team demonstrated promising results, with high rates of antibiotic completion and MOUD uptake, reduced patient-directed discharges, and lower readmission rates compared to historical controls. These outcomes highlight the potential efficacy of the integrated care model in managing PWID with SIRIs and addressing the broader syndemic of infectious diseases and SUDs. However, that pilot work was limited by non-randomized evaluation and study at a single hospital site. The multicenter randomized controlled trial design of the HI-SIRI study allows for a robust evaluation of the intervention’s effectiveness across diverse geographic locations and healthcare settings. By including sites with varying levels of access to SUD treatment and differing local health policies, the study aims to maximize external validity and generalizability.

One of the primary strengths of the HI-SIRI study is its focus on patient-centered care and adaptation to the needs of the individual. The intervention team tailors antibiotic treatments and SUD management to the individual needs of each participant, considering their social circumstances and preferences [49]. This approach is crucial for engaging PWID, who often face stigma, discrimination and barriers to accessing healthcare [18, 69–71]. The inclusion of harm reduction principles further supports the goal of reducing preventable morbidity and mortality without requiring abstinence from drug use [51]. In contrast to many SUD and infectious disease clinical trials, HI-SIRI will be testing a model of care, rather than a particular medication or specific behavioral therapy. The study will not directly pay for medical care/medications but rather will work within the existing healthcare structure to fund treatments using existing means. The study’s comprehensive implementation evaluation will provide valuable insights into implementation challenges of the integrated care model and potential implementation strategies for future testing, if the intervention is shown to be effective. Understanding these factors is essential for scaling up the intervention and ensuring its long-term success in various healthcare settings. Additionally, cost effectiveness evaluation of the intervention will help determine how this more intensive—and possibly costly—treatment model measures against potential healthcare cost savings of readmissions.

The SIRI team intervention personnel includes clinicians with dual expertise in infectious disease management as well as addiction medicine [41]. The addition of patient navigators, most of whom have lived experience with SUD, further strengthens the ability of the team to connect with a population that often harbors mistrust of healthcare providers. Team members will meet participants at the bedside and continue to build trust and support after hospital discharge, throughout the 4-month intervention period.

Further strengths include the unique patient population, broad substance use inclusion criteria, and a non-abstinence-based primary outcome. PWID hospitalized with infectious diseases have been underrepresented in broader infectious disease clinical trials. For example, the largest randomized trial in endocarditis included only 5 out of 400 patients with a history of IDU [72]. There is great interest in the ID community in implementing more diverse infection treatments, such as oral antibiotics for invasive infections and long-acting injectable antibiotics, for PWID, yet very little has been done in the way of prospective research to inform these approaches [73]. There is great interest in medical treatment of OUD, for which we have highly effective pharmacotherapy [74], but less data on approaches to management of infectious diseases in the face of stimulant use disorder, despite worse clinical outcomes [75]. Consistent with the fourth wave of the overdose crisis, we expect our cohort will include many with co-use of opioids and stimulants [76]. There will also be persons who only use stimulants, for whom SIRI treatments are understudied. In keeping with a trial rooted in harm reduction, the primary outcome does not rely upon any measures of substance use. The primary goal of the trial is to test whether the compassionate, nonjudgmental, patient-centered approach to SUD and infectious disease care can be successful in keeping people alive (the overarching goal of harm reduction) and out of the hospital during a vulnerable period.

Despite the promising pilot study results, success in the HI-SIRI study faces several challenges and potential limitations. The non-blinded nature of the trial may introduce bias, and the reliance on self-reported data for some outcomes could affect the accuracy of the findings; however, self-reported healthcare utilization will be validated against medical record abstraction. The intervention involves substantial contact with the medical team, which could be costly, especially physician involvement. While we do not expect any cross-over of TAU participants into the intervention arm, the development of local SIRI teams, clinician peer education, and leadership could bleed over into the TAU arm with potential to dilute the effect of the intervention. For example, a SIRI team might create an infectious disease screening order set that could start to be used by physicians caring for TAU participants, thereby improving infection screening in both treatment arms. The impact of having a SIRI team in the hospital will be further characterized in the implementation science component of the trial. Finally, loss to follow up is common in trials involving individuals with SUD and we expect even higher among PWID hospitalized with infections. In order to mitigate this risk, participants will only be enrolled if they provide at least minimum pre-defined locator information and participants with no phones will be provided with study phones. Furthermore, the primary outcome is available independent of whether a participant is lost to follow up.

If successful, the HI-SIRI study has the potential to significantly impact the care of PWID with SIRIs by providing a holistic, patient-centered approach that integrates infectious disease and SUD treatment with harm reduction strategies. The findings from this study will have the potential to transform our approach to care for PWID with infections, advancing health for a community long left behind in healthcare innovations.

## Data Availability

This study will comply with the NIH Data Sharing Policy and Implementation Guidance. Investigators will register and report results of the trial in ClinicalTrials.gov, consistent with the requirements of the Policy on the Dissemination of NIH-Funded Clinical Trial Information and the Clinical Trials Registration. Primary data for this study will be available to the public in the NIDA data repository, per NIDA CTN policy.

## Appendices:

A. SPIRIT 2025 checklist
B. Protocol 4.0
C. Model informed consent form

## Abbreviations

ART: Antiretroviral therapy
CAPI: Computer Assisted Personal Interviews
CCC: Clinical Coordinating Center
CHERISH: Center for Health Economics of Treatment Interventions for Substance Use Disorder, HCV, and HIV
DAA: Direct Acting Antivirals for Hepatitis C Virus
DSC: Data and Statistics Center
DSMB: Data and Safety Monitoring Board
ED: Emergency Department
EHR: Electronic Health Record
GEE: Generalized Estimating Equations
GLMM: Generalized Linear Mixed Model
HI-SIRI: Holistic Intervention for Severe Injection-Related Infections
HIV: Human Immunodeficiency Virus
HOPPER: Health and economic outcomes of treatment with extended-release naltrexone among pre-release prisoners with opioid use disorder
ICER: Incremental Cost-Effectiveness Ratio
ICU: Intensive Care Unit
ID: Infectious Diseases
IDU: Injection Drug Use
JCOIN: Justice Community Overdose Innovation Network
MAUD: Medication for Alcohol Use Disorder
MOUD: Medication for Opioid Use Disorder
NMOS: Non-study Medical and Other Services
PRISM: Practical, Robust Implementation and Sustainability Model
PWID: People Who Inject Drugs
QALY: Quality-Adjusted Life Year
SIRI: Severe Injection-Related Infection
SPIRIT: Standard Protocol Items: Recommendations for Interventional Trials
SSP: Syringe Services Program
SSTI: Skin and Soft Tissue Infection
SUD: Substance Use Disorder
TAU: Treatment As Usual

## Declarations

### Trial Registration

ClinicalTrials.gov NCT05688423 (https://www.clinicaltrials.gov/study/NCT05688423). Trial registry name: CTN-0121: Integrated Care and Treatment for Severe Infectious Diseases and Substance Use Disorders (SUD) among Hospitalized Patients. Trial Sponsor: Landhing Moran, PhD, landhing.moran@nih.gov, National Institute on Drug Abuse Clinical Trials Network. Registration date: January 17, 2023. Protocol Version 4.0.
